# Dietary Management of Heart Failure: DASH Diet and Precision Nutrition Perspectives

**DOI:** 10.3390/nu13124424

**Published:** 2021-12-10

**Authors:** Brooke E. Wickman, Byambaa Enkhmaa, Ronit Ridberg, Erick Romero, Martin Cadeiras, Frederick Meyers, Francene Steinberg

**Affiliations:** 1 Graduate Group in Nutritional Biology, Department of Nutrition, University of California, Davis, CA 95616, USA; bewickman@ucdavis.edu; 2 Center for Precision Medicine and Data Sciences, School of Medicine, University of California, Davis, Sacramento, CA 95817, USA; ebyambaa@ucdavis.edu (B.E.); raridberg@ucdavis.edu (R.R.); fjmeyers@ucdavis.edu (F.M.); 3 Department of Internal Medicine, Division of Endocrinology, Diabetes, and Metabolism, School of Medicine, University of California, Davis, Sacramento, CA 95817, USA; 4 Department of Internal Medicine, Division of Cardiovascular Medicine, School of Medicine, University of California, Davis, Sacramento, CA 95817, USA; esromero@ucdavis.edu (E.R.); mcadeiras@ucdavis.edu (M.C.)

**Keywords:** heart failure, dietary approaches to stop hypertension, DASH diet, diet pattern, metabolism, metabolomics, precision nutrition, personalized nutrition

## Abstract

Heart failure (HF) is a major health care burden increasing in prevalence over time. Effective, evidence-based interventions for HF prevention and management are needed to improve patient longevity, symptom control, and quality of life. Dietary Approaches to Stop Hypertension (DASH) diet interventions can have a positive impact for HF patients. However, the absence of a consensus for comprehensive dietary guidelines and for pragmatic evidence limits the ability of health care providers to implement clinical recommendations. The refinement of medical nutrition therapy through precision nutrition approaches has the potential to reduce the burden of HF, improve clinical care, and meet the needs of diverse patients. The aim of this review is to summarize current evidence related to HF dietary recommendations including DASH diet nutritional interventions and to develop initial recommendations for DASH diet implementation in outpatient HF management. Articles involving human studies were obtained using the following search terms: Dietary Approaches to Stop Hypertension (DASH diet), diet pattern, diet, metabolism, and heart failure. Only full-text articles written in English were included in this review. As DASH nutritional interventions have been proposed, limitations of these studies are the small sample size and non-randomization of interventions, leading to less reliable evidence. Randomized controlled interventions are needed to offer definitive evidence related to the use of the DASH diet in HF management.

## 1. Introduction

Approximately 6.2 million adults in the United States have heart failure (HF) [1]. The overall improved average survival of the population and the persistent incidence of cardiovascular disease (CVD) will increase the prevalence of HF and impair the functional quality of life of these individuals and their caregivers. Strategies for the effective management of HF are urgently needed. Though primary prevention of incident HF is also an important research topic, the scope of this review does not include HF prevention and instead focuses on the management of diagnosed HF.

HF is the end stage manifestation of various pathophysiologic disruptions of cardiac function characterized by insufficient cardiac output to meet body tissue metabolic demands [2]. HF does not affect all populations equally, and is a source of health disparities and inequities. Advances in medical management, as well as comprehensive nutrition and behavioral change interventions may synergistically impact heart failure pathophysiology and symptomology to improve patient health outcomes and quality of life. Though fluid and sodium restriction are often recommended for HF patients, the use of a comprehensive diet plan in diagnosed HF has not yet become standard of care in HF management.

Challenges to successfully managing HF include (1) diverse disease etiologies with varied responses to treatment regimens, (2) nutrition therapies inconsistent with patient preferences that reduce patient quality of life and adherence to dietary recommendations, and (3) metabolic heterogeneities affecting individual disease progression and management response. For instance, HF with preserved ejection fraction (HFpEF) and HF with reduced ejection fraction (HFrEF) are dissimilar in their responses to treatment. While HFrEF responds well to standard treatment, HFpEF is less responsive and new evidence-based care strategies are being tested.

Despite these challenges, there are also opportunities to better understand HF and reevaluate long accepted but non-individualized HF standards of care. Interdisciplinary research and health care teams are best equipped to monitor and interpret clinically relevant metabolic pathways and biomarkers while prioritizing functional patient-centered outcomes along with engaging HF patients, caretakers, and the community to effectively manage HF.

Improved outpatient disease management may slow disease progression and improve health outcomes such as re-hospitalization and death and are therefore important research objectives. Nutritionally, comprehensive diet approaches with individualized flexibility may meet the needs of HF patients better when compared to fluid and sodium restrictions. The Dietary Approaches to Stop Hypertension (DASH) diet is a promising combination diet that may be influential in HF management if it can be incorporated into the lives of HF outpatients. This comprehensive dietary approach allows more opportunities for individualization through precision nutrition and may be beneficial for successful HF management in free-living patients, rather than a reductionist focus on nutrient intake [3]. Coupled with behavior change techniques, the DASH diet may have positive effects in HF. Understanding the DASH diet’s clinical effectiveness and influence on metabolic biomarkers will be critical to build the scientific evidence base for precision nutrition recommendations and for inclusion as standard of care for outpatient HF management.

The purpose of this review is to summarize the literature related to the implementation and effectiveness of the DASH diet in outpatient HF management and to inform future precision nutrition research for cardiovascular health.

## 2. Methods

Available literature related to the DASH diet were reviewed for human studies without a time restriction. Relevant and reviewed studies included randomized controlled trials, prospective and cross-sectional studies, systematic reviews, and meta-analyses. Online literature was searched using PubMed. Search terms included heart failure, Dietary Approaches to Stop Hypertension (DASH diet), diet, diet pattern, dietary behavior, implementation science, metabolism, metabolomics, and outcome. Search term combinations included but were not limited to diet pattern and metabolism and heart failure, DASH diet and metabolism and heart failure, diet pattern and heart failure, diet patterns and heart failure and outcome, DASH and heart failure, and diet and heart failure. A total of 85 articles were identified after the initial search, while additional search terms and criteria were included as reviewers became increasingly familiar with the literature. Articles were organized by themes of diet pattern and HF; DASH diet and HF; and diet, metabolism, and HF.

## 3. Heart Failure Overview

### 3.1. Heart Failure

HF is the end stage manifestation of heart diseases, characterized by insufficient cardiac output to meet the body’s metabolic demands [2]. The impaired oxygen kinetics of HF contribute to poor quality of life with high rates of morbidity and mortality associated with HF [4]. Symptoms that particularly impact activities of daily living include shortness of breath, fatigue, and edema. The clinical syndrome of HF can be the result of various etiologies that affect the myocardium, cardiac valves, pericardium, or vessels. Co-morbid risk factors for HF include smoking, hypertension, obesity, diabetes or insulin resistance, and ischemic heart disease.

HF is a heterogeneous syndrome where each patient’s complex characteristics dictate the functional and structural biomarker changes during disease progression. HF is classified into four sequential stages where A (at risk) is at risk for HF due to pre-existing conditions; B (pre-HF) is asymptomatic structural heart disease; C (HF) is symptomatic structural heart disease; and D (advanced HF) is end stage HF with marked symptoms at rest despite therapeutic interventions [5]. HF can also be classified into categories based on left ventricular ejection fraction (LVEF): HF with reduced ejection fraction (HFrEF), HF with mildly reduced ejection fraction (HFmrEF), and HF with preserved ejection fraction (HFpEF) [5]. The LVEF-based classification is somewhat arbitrary and has imprecise physiological implications, as there are almost always overlapping phenotypes and the pathophysiology is intertwined [6]. HF pathophysiology and neurohormonal activation have been extensively reviewed elsewhere [7,8,9].

### 3.2. Prevalence and Importance of Interventions

**Figure 1:**
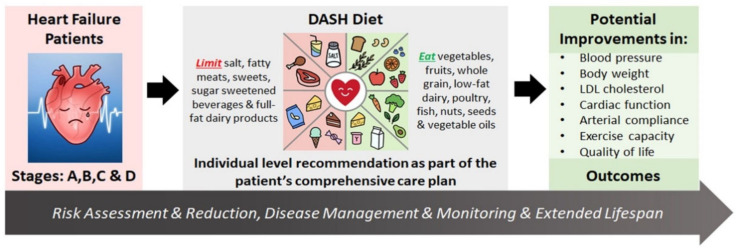
The potential role of the DASH diet in heart failure management. Assessment of patients with heart failure (HF) stages (A–D) is followed by comprehensive clinical care management, which includes an individual-level DASH diet recommendation. Once successfully adopted and adhered to, the DASH diet can improve patients’ physical and functional capacities through reductions in blood pressure, body weight, and LDL cholesterol concentration and improvements in cardiac function, arterial compliance, exercise capacity, and quality of life. The positive effects of the DASH diet implemented as part of a comprehensive care plan for risk reduction and disease management and monitoring can contribute to improved health outcomes.

With over 6 million Americans and over 26 million people worldwide diagnosed with HF, the prevalence continues to rise with survival improvements and population aging, while large increases in HF incidence are expected in older adults of all sex and racial/ethnic groups through the year 2030 [1,10,11]. In the US, the economic burden of the direct and indirect costs of HF in 2012 was $30.7 billion, with 80% of the medical costs of HF related to hospitalization [11,12,13]. Strategies for HF prevention and management are critical. Dietary and behavioral interventions that have demonstrated efficacy in reducing risk factors and co-morbid conditions, such as the DASH diet (Figure 1), are amenable to individualization and adaptation to fit a wide variety of tastes and cultural patterns and are important strategies to mitigate HF prevalence and burden [5,6,7].

## 4. Current Cardiovascular Disease Risk Reduction Recommendations

Diet patterns that support good health emphasize the consumption of fruits, vegetables, whole grains, lean protein sources, legumes, dairy, nuts, and healthy fats, while limiting intake of energy-dense sugars and processed foods. The DASH diet has been shown to be efficacious for CVD risk reduction and is a healthy diet pattern endorsed in the USDA Dietary Guidelines for Americans, 2020–2025 [14,15]. While other dietary patterns are included in these guidelines, the composition of comprehensive diet practices such as Mediterranean and vegetarian diets have been variably defined in the literature, making their systematic evaluation more challenging. The DASH diet is one of several included in the American Heart Association/American College of Cardiology (AHA/ACC) recommendations for diet patterns that can help adults who need to lower low-density lipoprotein cholesterol (LDL-C) and blood pressure (BP); the DASH diet is especially noted for the extensive evidence to that effect [16]. The US Preventive Services Task Force 2020 recommendations include behavioral counseling interventions to promote a healthy diet and physical activity for all adults with CVD risk based on evidence of moderate net benefit [17]. Globally, the European Society of Cardiology guidelines recommend that HF patients receive education on fluids to avoid excessive fluid intake and dehydration and on a healthy diet to prevent malnutrition, avoid consuming >5 g of salt daily, and healthy body weight maintenance [18]. Though several expert panels have endorsed a multifaceted lifestyle approach combining diet, exercise, and pharmacological therapy in order to promote improved HF outcomes (Figure 1), widespread implementation of these sensible and effective measures has been elusive.

## 5. Dietary Approaches for Heart Failure Management

### 5.1. Sodium and Fluid Restriction

Nutrition care is integral to comprehensive HF management. The current primary dietary approaches for HF management are sodium and fluid restriction, but individualization to patient needs is essential as multiple variables need to be considered in advanced HF [19,20]. Various levels of sodium restriction may be recommended in clinical practice, despite limited rationale for these levels. In the 1997–1999 DASH–Sodium trial, three sodium levels were set for diets based on the typical US sodium consumption (3450 mg/d), the recommended upper limit of sodium intake at the time of the study (2300 mg/d), and potentially optimal sodium intake levels (1500 mg/d) [21]. When combining a low sodium intake of 1500 mg/d with the DASH diet compared to a 3450 mg/d high sodium intake from a control diet, BP was lowered 7.1 mmHg for those without hypertension and 11.5 mmHg in participants with hypertension, though BP reductions were observed with lower sodium intake independent of diet type [22]. Limiting sodium intake to 1.5 g/d requires consumption of reduced sodium and no salt added versions of most foods with restricted salt use with food preparation. Additionally, potential barriers to achieving low sodium adherence of 1.5 g/d include awareness and availability of reduced sodium food options, familiarity with alternative flavoring options when preparing foods, and food palatability and preferences. Sodium intake of 2.3 g/d is more achievable than lower sodium levels with regular foods, avoidance of highly salted processed foods, limiting use of table salt, and modestly seasoning with salt while cooking. Even when reduced sodium intake is achieved, however, not all individuals experience BP changes in response to sodium intake, indicating people can be salt-sensitive or salt-resistant [23].

Factors to consider when personalizing sodium recommendations for HF patients include HF stage and symptoms, LVEF, diagnosis of HFpEF vs. HFrEF, concurrent medications affecting cardiac function and urinary output, comorbidities, patient body size, and baseline diet. While excessive sodium and fluid intake seen in the setting of non-compliance are common causes of HF exacerbation and hospitalizations, overly strict recommendations can have adverse nutritional and physiologic consequences. Excessive sodium and fluid restrictions increase the perception of thirst [24]. Severe sodium restriction may result in reduced palatability and intake overall, thus increasing the risk of nutritional inadequacies and deficiencies [20,25]. In contrast to strict low sodium recommendations, modest sodium may contribute to cardiac performance in compensated HF, and the level recommended should be based on clinical evaluation of the patient [26]. A recent systematic review evaluating dietary interventions consisting of 1.5–3 g of daily sodium intake did not find robust or conclusive support of efficacy for a particular dietary sodium recommendation [25]. Professional society guidelines typically endorse a 2–3 g/d sodium intake depending on HF stage based on fair or level C strength of scientific evidence and is a recommendation consistent with public health recommendations for chronic disease prevention [20,27].

For fluid restriction, the ACC/AHA HF guidelines suggest fluid restriction of 1.5 to 2 L/day in patients with HF class D or severe hyponatremia [27]; the recommendations are based on expert opinion due to the limited evidence on the benefits of such recommendations [28,29]. Beyond this, the cardiology societies do not provide comprehensive dietary recommendations or guidelines. There is room for additional scientific evidence regarding the efficacy and effectiveness of optimal diet pattern and composition recommendations in HF patients and best practice approaches to provide individualized care to improve clinical outcomes.

### 5.2. Body Weight Management

#### 5.2.1. Overweight, Obesity, and Underweight

Excess body weight results in metabolic and neurohormonal pathways that promote cardiac remodeling, risk of HF, and incidence of HF symptomology [30]. Weight reduction through various means improves cardiac function, symptom burden, and potentially HF hospitalizations [31,32,33,34,35,36]. This is particularly true in overweight and obese middle-aged patients with stage C HF, and may prevent further progression of stages A-B HF. In contrast, there is a U-shaped relationship between BMI and mortality for patients with HF [37,38,39] with some excess weight the most protective in females with advanced HF of stages C-D [40]. Underweight status including obese or lean sarcopenia is associated with advanced HF and is prognostic of poor outcomes. Cardiac cachexia is an end-of-life indicator in the trajectory of HF illness.

#### 5.2.2. Inadequate Intake and Malnutrition

Chronic inadequate caloric intake in HF patients leads to sarcopenia and cachexia, ultimately increasing the risk of mortality and recurrent hospitalizations. Inadequate intake has been found to be an independent predictor of adverse events, even when confounding factors are controlled [41]. Micronutrient deficiencies may be due to poor diet quality or to inadequate intake of both micro- and macronutrients, existing across BMI categories and associated with increased risk of morbidity and mortality [20,42]. Healthy diet patterns such as the DASH diet provide nutrient-dense patterns, but successful strategies for intensive nutritional support of individuals with HF and significant malnutrition are beyond the scope of this review.

### 5.3. Dietary Patterns

Dietary quality in the context of various healthy diet patterns is an important determinant of health outcomes. Various dietary quality scores have been used to evaluate eating patterns, such as the Healthy Eating Index-2015 (HEI-2015), Alternate Healthy Eating Index (AHEI), Healthful Plant-Based Diet Index (HPDI), and Alternate Mediterranean Diet Score (AMED). High adherence to each of these scores was consistently and inversely associated with CVD risk in large prospective study populations with long-term follow-up [43]. This underscores recommendations for individualization, personal preference, and cultural choices in adapting a variety of eating patterns for health promotion. Healthy eating patterns such as the DASH and Mediterranean diets share many common characteristics (rich in fruits, vegetables, whole grains, legumes, and nuts) while still retaining distinctive qualities through specific foods or combinations of foods.

The Mediterranean diet and the DASH diet are well documented to be protective against the incidence of CVD [44]. In efforts to assess a full diet approach for HF management, a predominately plant-based dietary pattern, as well as the DASH and Mediterranean diet patterns, have been identified as protective against HF incidence and preserve cardiac function [45,46]. However, the DASH diet appears to be more consistently successful compared to the Mediterranean diet [47,48]. The Mediterranean diet is the only pattern assessed for risk of HF in a large-scale RCT in high-risk individuals, and while it did not demonstrate a statistically significant reduction in HF incidence, the respective study may have been underpowered for this outcome [49].

#### The DASH Diet

**Figure 2:**
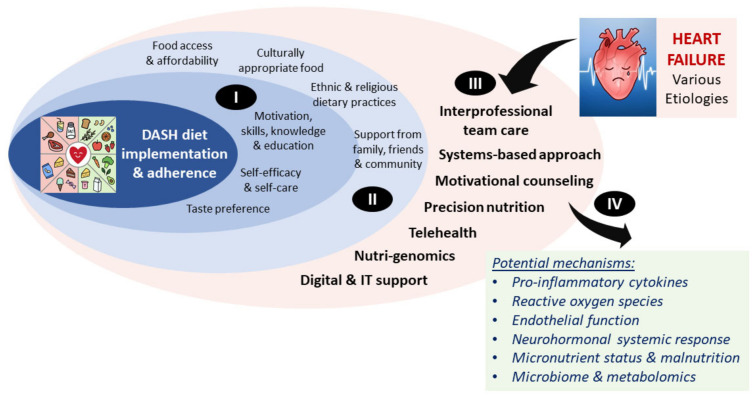
Potential predictors of DASH diet success and mechanisms underlying its positive effects in heart failure. The DASH diet effectiveness in HF risk reduction and management can be influenced by many different factors. Examples of individual patient-level factors (I) and factors associated with the environment, society, and culture (II), which are also intertwined with the individual patient-level factors, are shown. In addition, there are factors specifically associated with the clinical management of heart failure that may influence DASH diet success (III) Once successfully implemented and adhered to, the DASH diet may reduce HF risk through several different mechanisms associated with improved systemic and local responses (IV).

The DASH diet emphasizes the consumption of whole grains, fruits and vegetables, low-fat dairy, lean meat, fish, poultry, nuts, seeds, and legumes, and sparse use of fats and oils (Figure 1). This eating pattern is high in antioxidants, micronutrients, fiber, and nitrates while low in saturated and trans fats (Appendix A, Table A1) [50]. This synergistic dietary pattern of nutrients is suggested to address some of the underlying pathophysiology of HF through decreased pro-inflammatory cytokines and reactive oxygen species, promoting endothelial function, restoring micronutrient status, and combating malnutrition [51]. Implementation and adherence impact the effectiveness of the DASH diet and sodium reduction in HF management, but other determinants, such as social and cultural norms, social support, food access and affordability, taste preferences, and self-efficacy, may be predictive of dietary intervention success [52]. This complex interplay is depicted in Figure 2.

Hypertension is a risk factor for HF incidence as well as a target for HF management, and BP reductions can be achieved with the DASH diet (Appendix A, Table A1). The original DASH study by Appel et al. showed that controlled feeding of a comprehensive combination diet high in fruits, vegetables, and low-fat dairy and low in saturated fat, total fat, and cholesterol reduced systolic BP by 5.5 mmHg and diastolic BP by 3.0 mmHg more than a control diet, while a fruit- and vegetable-rich diet resulted in BP reductions of 2.8 mmHg systolic and 1.1 mmHg diastolic BP more than a control diet, with greater reductions observed in those with hypertension [53]. Though several studies have evaluated DASH diet interventions for BP changes, consistency among diets is difficult to assess due to variable sources used for diet nutritional analyses methods (menu vs. intake nutrient analyses) and variability in nutrient analysis transparency (Appendix A, Table A1).

The BP reductions that can be achieved with the DASH diet are important in HF pathophysiology, but the protective effects likely extend beyond BP control. The DASH diet is beneficial in preventing the incidence of HF in patients younger than 75 years as reported from the MESA (Multi-Ethnic Study of Atherosclerosis) [54]. This conclusion was reinforced in findings from the large and diverse REGARDS (the REasons for Geographic and Racial Differences in Stroke) cohort, which demonstrated benefit from moderate through high quintiles of DASH adherence [55]. The DASH diet reduces BP with or without reducing sodium intake, though greater BP control is achieved with sodium reduction [22].

### 5.4. Nutritional Supplements and Treatments

Dietary supplements have been studied for their effects on HF management and have proven ineffective or not yet reproducible. Antioxidant vitamins (such as vitamin C and E) have been largely ineffective in clinical trials, while coenzyme Q10 (CoQ10) has been studied as a targeted approach to treat oxidative stress and mitochondrial dysfunction, with modest yet controversial results [56,57]. Results from a global CoQ10 adjunctive treatment trial indicated lower cardiovascular mortality, all-cause mortality, and HF hospital stay incidence in patients with moderate to severe HF compared to placebo control [58]. Inconsistency in the literature is demonstrated by two meta-analyses of CoQ10 supplementation trials reporting improved left ventricular function and another concluding no significant differences exist, fueling continued interest in the topic [59]. A recent meta-analysis of 12 randomized trials evaluating marine omega-3 supplements in HF found conflicting results across trials but showed a significant reduction in recurrent HF hospitalizations compared to placebo and no effect on cardiovascular mortality [60]. Treatment of patients with chronic HF and iron deficiency with oral supplementation is not included in HF guidelines in the absence of iron deficiency anemia, whereas using intravenous ferric carboxymaltose compared to placebo reduced the risk of recurrent HF hospitalizations in a large multicenter trial [61].

## 6. Physiological Mechanisms of the DASH Diet in Heart Failure

The DASH diet is effective in the prevention of CVD. However, the effects of the DASH diet on the routine medical management of free-living HF patients are less understood. Consistent observational evidence supports DASH diet eating patterns are associated with improved cardiac function, lower HF event rates, and reduced mortality [62]. There is some evidence from a small intervention trial that the DASH diet may protect against disease progression in those diagnosed with HF, especially hypertensive HFpEF [63]. Systematic reviews provide further evidence of the protective role of the DASH diet pattern in CVD, including HF [15,47,64]. DASH diet adherence, possibly due to the dietary components and low sodium intake, is beneficially associated with BP reductions and left ventricular function in HF [45].

### 6.1. Potential Mechanisms

Evidence supports that the DASH diet may improve cardiac function, functional capacity, blood pressure, oxidative stress, and mortality [47]. Additionally, the DASH diet has been shown to improve arterial compliance, exercise capacity, and quality of life [65]. Data from the large MESA study demonstrated a positive association between an increase in DASH diet adherence score and improved cardiac function assessed by end diastolic volume and stroke volume [66]. The DASH diet is low in sodium and high in potassium, a combination that has been shown to reduce BP [67]. Additionally, the high magnesium, calcium, nitrates, and antioxidants in the DASH diet compared to unhealthy diets may contribute to cardiovascular health benefits [47]. The DASH diet is an overall eating pattern rich in unsaturated fats and plant-based foods, providing a complex mixture of nutrients, fiber, and bioactive diet components with multiple physiological targets and intermediate biomarker effects [65,68,69,70]. The DASH diet’s protective mechanisms may result from synergistic effects in various physiological pathways related to antioxidant capacity, inflammation, liver function, coagulation, natriuresis, sympathetic activation, and endothelial function [45]. Furthermore, very little is known about the dietary modulation of the gut microbiome that may occur in the setting of dysbiosis and chronic gut hypoperfusion of end stage HF [71].

### 6.2. Specificity and Efficacy Depending on Type of Heart Failure Diagnosis

HF is a set of complex and heterogeneous diagnoses with underlying variability in etiology, pathophysiology, metabolic, and other individual factors. HFrEF is responsive to a standardized treatment approach despite heterogeneity of etiologies [27]. Conversely, HFpEF is not as efficiently treated and may be treated more effectively with targeted approaches based on advanced phenotyping [72]. Phenotyping has the potential to identify subgroups with differential responses to therapy and cohorts for personalized interventions in HFpEF [73].

The taxonomy or classification of HF recognizes the diverse etiologies and associated mechanistic disruptions of cardiac function. Biomarkers that predict progression or response to treatment will aid in the development of a new precision nutrition taxonomy that will identify those patients most likely to benefit from the DASH diet. The inverse association of DASH diet adherence with incident HF in the REGARDS cohort was not statistically different between HFrEF and HFpEF [55]. A recent study demonstrated significant positive effects of a DASH sodium-restricted diet via two distinct cardioprotective mechanisms. Compared to control diet, a DASH diet alone decreased a biomarker of cardiac injury, high sensitivity cardiac troponin1 (hs-cTn1), while a sodium-reduced diet decreased a biomarker of cardiac strain, N-terminal b-type pro natriuretic peptide (NT-proBNP). A DASH diet combined with modest sodium restriction significantly decreased hs-cTn1 by 20% and NT-proBNP by 23% [74]. These results are analyses from the original DASH study population with untreated hypertension and no documented CVD who were provided highly controlled study meals. It is unknown whether these mechanisms and clinical biomarkers will be similarly responsive in diagnosed HF patients with and without preserved ejection fraction (HFpEF vs. HFrEF).

### 6.3. Metabolic and Metabolomic Biomarkers

Studying specific metabolites with metabolomics techniques has been used to further understand HF and the DASH diet. The innovative use of metabolomics profiling in nutrition research is growing and can identify metabolites as biomarkers of dietary pattern and food exposure to advance the ability to evaluate dietary adherence and nutritional intake assessment in a quantitative manner [75]. Additionally, metabolomics can interrogate metabolic pathways and disease biomarkers to characterize systemic changes in metabolism associated with dietary intake of medical management and health. Metabolomic techniques characterize small molecules and metabolites in biologic fluids such as blood and urine and can expand our understanding of the bi-directional relationships between diet and nutrition and the physiologic responses important to health and disease. This exciting scientific advancement in the field of nutrition research carries a great deal of complexity and numerous challenges to be considered and resolved with the conduction, interpretation, and comparison across studies of such research. Aspects contributing to this include heterogeneity in study designs and sampling procedures; biologic variability in response to foods and food components; analytical platforms and methods used for metabolite detection; limitations of current metabolite databases including robust, sensitive, and specific validated food biomarkers and their temporal biologic responses, and; various biostatistical methods used [76].

The medical literature includes multiple studies associating metabolites with HF characteristics and outcomes. Examples include findings that metabolic profiling can discriminate and identify patients at risk of HF-related decompensation, and metabolomic profiles showing differentially expressed signaling pathways are associated with lower cyclic guanosine monophosphate/brain natriuretic peptide (cGMP/BNP) ratios in HF patients [77,78]. Of relevance to the diet and gut health relationship, the gut-related metabolite trimethylamine N-oxide (TMAO) is associated with acute and long-term adverse outcomes in HF [79].

Several observational and cross-sectional studies have identified dietary-derived serum metabolites characteristically associated with specific foods and healthy diet patterns, including the DASH diet [80,81,82,83,84]. Healthy eating patterns are comprised of some similar foods and food components, which is seen in the overlap of metabolites reflecting complex dietary patterns. Unique metabolites associated with the DASH pattern have been described in several populations [82]. Seven interventional studies have evaluated dietary-responsive metabolites in DASH feeding trials (Table 1 and Appendix B Table A2). Serum samples from the original DASH feeding trial in subjects with pre-hypertension were analyzed in untargeted protocols and 44 metabolites were identified that were significantly different between the DASH and either control or fruit and vegetable patterns, with 10 metabolites that were proposed as a candidate panel of biomarkers for DASH diet adherence [85]. Metabolomic analyses of the DASH–Sodium trial were conducted by three groups, which identified metabolite pathways that were altered and associated with differing levels of sodium intake, which may be reflective of differing food and herb choices related to the lower sodium diet, intake of salt itself, as well as the metabolic profiles characteristic of salt-sensitive subjects [86,87,88]. The DASH–Sodium trial samples were also used to evaluate metabolic pathways unrelated to diet exposure but instead representative of sleep habits [89].

Two recent small, controlled feeding trials of the DASH pattern demonstrated metabolites specific to the DASH diet and food-specific components reflecting the intervention diet [91,92]. In a HFpEF study using targeted metabolomics, a controlled DASH sodium-restricted diet resulted in metabolite changes correlated with ventricular contractility and inversely with ventricular stiffness, suggesting metabolically beneficial effects due to dietary modifications [90]. These findings need to be replicated due to the small sample size and single arm study design. A metabolomics study of chronic kidney disease (CKD) patients demonstrated the ability to detect metabolic signatures associated with healthy diet patterns, such as DASH, demonstrating proof of principle for use in a free-living patient population with chronic disease [93]. Food-specific compounds can be cataloged with metabolomics and detected in urine after a dietary intervention, and these techniques have been successful when DASH-style foods were used as the intervention [91]. A significant gap in the existing literature is the absence of studies using metabolomics to identify adherence to the DASH diet and associated metabolic effects in a free-living HF patient group rather than controlled dietary feeding trial.

## 7. DASH Interventions in Heart Failure: Food/Nutrients and Education Interventions

### 7.1. Controlled Feeding Trials

Clinical trials are contributing to emerging evidence that the DASH diet is efficacious in HF management. A three-week diet intervention of DASH sodium-restricted meals provided to HFpEF patients was associated with significant reductions in BP and arterial stiffness, improved ventricular diastolic function, and reduced oxidative stress [63,94]. This small pre-post pilot study demonstrates proof-of-concept for use of the DASH comprehensive diet approach in this population. When the DASH diet was administered directly to discharged patients via home-delivered meals, the GOURMET-HF study showed that recently hospitalized HF patients had improvements in symptoms, physical limitations, and hospitalizations [95]. The effectiveness of the DASH diet nutrition education and restrictive meals needs evaluation in a pragmatic HF outpatient care setting, with goals to replicate the promising findings of the controlled diet studies and to empower patients through education and motivational interviewing counseling to actively manage their disease through dietary modifications and lifestyle change actions.

### 7.2. Behavioral and Lifestyle Interventions

Examples of successful DASH lifestyle interventions include the PREMIER study and ENCORE trial, which resulted in the intervention groups significantly increasing consumption of nutrient-rich DASH-specific foods and food categories as compared to the advice only control group [96,97]. These studies utilized intensive group and individual counseling sessions (weekly, biweekly, and monthly follow-up for 4 to 18 months total) in hypertensive patient populations. Few studies have applied similar approaches to the HF population. A notable example is a 6-month randomized intervention in chronic symptomatic HF patients comparing DASH to usual care, which demonstrated positive effects of DASH with significantly improved exercise capacity and quality of life scores, along with a trend toward improved endothelial function [65]. Adherence to dietary sodium restrictions in chronic HF patients based on self-report and 24 h urinary sodium excretion is poor, and restrictive sodium interventions typically did not improve clinical outcomes [98,99]. This provides additional rationale for a focus on comprehensive dietary approaches such as the DASH diet.

### 7.3. Behavior Change Strategies

Sustaining behavior change around diet and other lifestyle habits is challenging for free-living outpatients with chronic disease. Assessment of adherence to HF self-care recommendations that include diet, medications, exercise, and other self-reported behaviors have revealed typically low or selective adherence [100]. Poor adherence is a contributing factor to high hospital readmission rates in HF patients [101,102].

Patient self-efficacy is a central precept in behavior and behavior modification. Interventions designed to improve self-efficacy should use validated tools to measure self-efficacy pre- and post-intervention, and the intervention should be targeted to improving self-efficacy to achieve dietary behavior change. Self-efficacy can be enhanced through a variety of evidence-based strategies utilized by registered dietitians and other healthcare providers, such as individualization to tailor recommendations, social support, goal setting, self-monitoring, and others [16]. Cognitive strategies such as motivational interviewing and cognitive behavioral therapy (CBT) may improve patient self-efficacy for initiating and maintaining behavioral diet changes. CBT improves self-care and quality of life in HF patients and could be applied to dietary counseling for DASH diet modifications [103]. An individually tailored behavioral intervention based on the transtheoretical model achieved significantly greater improvement in DASH diet score compared to usual care intervention in uncontrolled hypertensive patients [104].

Utilization of telemedicine can help control diseases, manage clinical cases, and support caregivers [105]. During the coronavirus disease-19 (COVID-19) pandemic, telehealth services reduced COVID-19 transmission risk while allowing continuation of required healthcare services for disease management [106]. CHF outpatients are able to adhere to telehealth protocols, but it is unclear whether telehealth adherence reduces hospitalizations and CHF readmission rates [107]. In a randomized controlled trial of the effectiveness of telephone-based health coaching for HF patients, unhealthy nutrition behaviors trended toward reduction without significant results [108]. Though there is limited evidence to support the use of telehealth nutrition counseling for HF management, telehealth dietary coaching was effective and feasible for stage 3–4 CKD management [109]. Whether digital health and telehealth services can be effectively used to support diet adherence in HF outpatients remains unknown, although trials are underway in other domains of HF self-management [110].

## 8. Implementation Analyses

Despite the importance of translating research findings into clinical care, it takes an estimated 17 years to incorporate evidence into practice [111]. There are many reasons for this delay, but failure to develop systems to support the identification, implementation, and evaluation of outcomes of local efforts is most prominent. Effective execution of health care delivery science requires team members with varied backgrounds and a support network of institutional partners [112]. Implementation science or systems-based practice promotes the inclusion of research results into practice with subsequent evidence-based assessments to improve the quality of health care [113]. Implementation science approaches are used to translate research effectively and swiftly into patient care to support the inclusion of new knowledge into systems of care to optimize health care and public health [114,115]. For example, the GOURMET-HF study demonstrates that home-delivered DASH diet meals are safe and effective for HF short-term discharge outcomes, however, sending outpatients home-delivered meals has not been practical for widespread implementation into the standard of care [95]. Adherence and efficiency outcomes can be used to assess nutrition intervention effectiveness in the pipeline of implementing evidence-based practices into health care [116].

Recognizing the potential of implementation science in nutrition research, the Academy of Nutrition and Dietetics identified implementation science as one of four domains for the organization’s 2020 research priorities [117]. An implementation science approach will maximize the impact of a dietary intervention for a clinical population by determining if the DASH diet can effectively be incorporated and sustained as standard of care for HF patients using controlled implementation trials. As the field of implementation science matures, evidence and guidance for using implementation science to improve nutrition outcomes will expand [118]. Understanding health systems by leveraging existing data while designing alternative health care practices is a priority in health care delivery science [119].

Along with efforts to focus on implementation of evidence-based strategies into clinical care through healthcare delivery science, clinicians and scientists must consider ways to reduce health inequities while implementing new care strategies. Recommendations to confront health inequities include developing networks of collaborations and stakeholders to launch transformative studies, optimizing new data sources, platforms, and natural experiments, developing transdisciplinary training programs to enhance research capacities, embracing inclusive research themes, and developing platforms for innovative transdisciplinary research promoting systems science approaches [120].

## 9. Opportunities for Precision Nutrition and Health in Heart Failure

A precision medicine taxonomy may enhance our understanding of the risk of HF and define interventions more likely to be effective or less toxic based upon the integration of many sources of data including phenotype, omics biomarkers, environmental, and behavioral domains. The variation is best understood by large data bases or cohorts that promote health equity by being inclusive and thus promote tailored therapies for all patients or persons at risk. Comprehensive molecular information, obtained by omic technologies, enables the conduction of precision medicine approaches. Precision nutrition, a form of precision medicine, promises a better understanding of variability in an individual’s response to dietary interventions. Specifically, precision nutrition is nutritional recommendation and guidance designed to maximize therapeutic benefit and health outcomes based on individual profiling or phenotyping. This in-depth metabolic and physiologic information is generated through genomic, metabolomics, proteomic, and metagenomics data. Examples include single nucleotide polymorphism genetic variations that impact nutritional needs or metabolic responses to diet, as well as the influence of gut microbiome composition as a determinant of glycemic and triglyceride responses [121,122,123,124,125]. The development of more precise and dynamic nutritional interventions is a key focus of the National Institutes of Health’s Nutrition for Precision Health Strategic Plan 2021–2025 [126]. The effective application of precision nutrition must consider individual and population level cultural context, dietary patterns, personal preference, and behavioral aspects that sustain healthy eating patterns and lifestyles. Precision nutrition can be paired with patient-centered care to test the idea that more partnerships with community stakeholders and multi-domain assessment of patients and their families will guide targeted interventions that consider the mechanistic variation noted above and the diversity of patients and communities served in healthcare. This diversity includes, but is not limited to, cultural beliefs about food and nutrition, behavioral support to modify consumption such as reduced salt supplementation of meals despite taste alterations, and financial insecurity that limits choice of foods. Patient-centered outcomes research that includes strong community engagement and partnerships will ensure that the needs of HF patients, families, and caregivers are addressed.

As noted, HF is a complex and heterogeneous condition involving various etiologies and dysfunctions across multiple organs, yet current nutritional care and recommendations focus on sodium, fluid, and the caloric intake of heart-healthy choices applied in a broad manner. The new taxonomy of HF promotes a targeted and personalized approach to clinical care [7,73,127]. Dietary interventions are typically described in terms of average (mean) responses, but biologic interindividual variability is high with a large distribution of responses [128]. Opportunities such as those described in the metabolomics section promise to generate data and gain a deeper understanding of variability within human biologic systems and molecular pathways that interact with and mediate relationships among dietary patterns, food components, social and environmental factors, genetics and host metabolic variability, gut microbiome, health, and disease status. The use of these data through integrative approaches and machine learning has the potential to predict and objectively measure patterns of response to nutrition interventions and food choices, such as responder and non-responder differences, to enable healthcare providers the choice of targeted and efficacious nutritional guidance for patients. For example, genetic information in the form of a specific single nucleotide polymorphism coupled with diet adherence have indicated significant stroke risk reduction [129]. Additionally, microbiome-derived metabolites in the gut, such as short-chain fatty acids, bile acids, TMAO, and amino acid metabolites, are affected in the functional host-microbe relationship and may be assessed with a personalized medicine approach for risk stratification of HF [130]. Pertinent goals to inform this objective include characterizing HF patient response to the DASH diet across phenotypes (such as HFpEF vs. HFrEF) and other metabolic biomarkers, evaluating the effects of DASH intervention on medical management, evaluating unbiased markers of food intake and metabolic nutritional assessment, and exploring behavioral and implementation science approaches to initiate and sustain positive eating behaviors of the DASH diet. Evidence-based nutrition recommendations for HF patients will allow for targeted and nuanced clinical nutrition care for individuals from diverse backgrounds with diverse HF diagnoses [126].

## 10. Summary and Recommendations

Implementation of evidence-based nutrition strategies that have shown benefit in carefully controlled clinical trials may positively impact HF outcomes in a cross section of the HF population. Use of the DASH diet in diagnosed HF patients has not yet become standard of care in HF management as it has not been evaluated in pragmatic clinical trials. There is a need for a revised taxonomy of HF that integrates the diverse causes of HF with the diverse demographics of patients with HF and the response or resistance to the DASH diet [51].

Additionally, health care implementation science approaches may ensure that combining effective nutrition interventions with systems of care that integrate behavioral support and modification can be successfully incorporated into routine clinical care. The stratification of patients with pre-existing self-efficacy and systems that support or improve self-efficacy are important baseline measurements. The ability of HF patients to successfully adopt the DASH diet outside of controlled feeding studies has not been widely studied. Precision nutrition technologies should also be explored to understand patient responsiveness to dietary interventions based on patient and disease characteristics such as genotype, HF etiology, therapeutic medications, and other parameters, along with omic technologies that enable objective assessments of dietary intake and subtle metabolic changes. Research studies evaluating the effectiveness and implementation of the DASH diet for outpatient HF management are warranted and have the potential to influence the future health care approaches in CVD prevention, treatment, and management.

## Figures and Tables

**Table 1:** Metabolite markers associated with DASH feeding studies.

Study	Aim	Design & Diet	Subject Group	Omic Method	Bio-Sample	Main Finding
Mathew A, et al., 2015 J Card Fail [90]	Characterize metabolomic changes following DASH Sodium-restricted diet intervention	Single arm, Controlled feeding study, 3 weeks DASH sodium reduced diet Ş	Adults with HTN and HFpEF (*n* = 13), predominately female	GC-MS and LC-MS, targeted	Serum	Short-chain acyl carnitine metabolites increased significantly, correlated with improved cardiac function
Derkach A, et al., 2017 AJCN [86]	Evaluate the effect of sodium intake on metabolomic profiles following control and DASH diet intervention	RCT, Crossover Controlled feeing study, 2-week run-in, 12 weeks DASH vs. Control arms each with 3 sodium levels for 4 weeks each intervention ¥	Adults with HTN, M + F, mixed race, *n* = 119 from DASH-Sodium trial; Samples compared high-versus low or medium sodium, independent of diet arm	LC-MS, untargeted	Plasma	Sodium intake significantly associated with changes in 6 metabolite pathways. Adjustment for change in diet pattern or blood pressure did not alter associations
Rebholz CM, et al., 2018 AJCN [85]	Identify metabolites associated with DASH diet pattern	RCT, Parallel, Controlled feeding study, 3-week Control run-in, 8 weeks feeding DASH diet or Control or Fruit & Vegetable-rich diet ≠	Adults with pre-and stage 1 HTN, M + F, mixed race, *n* = 329 (control 108, fruit/veg 111, DASH 110)	GC-MS and LC-MS, untargeted	Serum	Multiple metabolites differed significantly DASH vs. Control (97) and DASH vs. fruit/veg (67); Identified 10 most influential metabolites as candidate biomarkers for assessing adherence to DASH diet; metabolites represented classes of lipids, amino acids, xenobiotics and food components, cofactors and vitamins, carbohydrates
Reisdorph N, et al., 2020 Sci Report [91]	Characterize changes in metabolome following DASH diet intervention, and characterize food specific compounds (FSC)	RCT, Crossover, Controlled feeding study, 2-week habitual diet run-in, 6-week feeding each of two DASH style diets Ş	Adults, M + F, *n* = 19	LC-MS, untargeted	Urine and foods	FSC from DASH diet were detected in urine along with other metabolites; 16 metabolites were significantly associated with BP and 6 with change in BP
Chaudhary P, et al., 2021 Hypertension [88]	Evaluate the effect of sodium intake on metabolomic and lipidomic profiles between salt-sensitive and salt-resistant individuals	RCT, Crossover Controlled feeing study, 2-week run-in, 12 weeks DASH vs. Control arms each with 3 sodium levels for 4 weeks each intervention ¥	Adults with HTN, M + F, mixed race, *n* = 191 (106 salt-sensitive, 85 salt-resistant) from DASH-Sodium trial; Samples compared high-versus low sodium on Control diet arm	LC-MS, untargeted	Plasma	Baseline comparisons exhibited no differences, but 3 metabolites differed significantly in salt sensitive subjects with change in sodium intake
Kim H, et al., 2021 MNFR [87]	Determine if candidate serum biomarkers from original DASH trial replicate in urine in DASH-Sodium trial	RCT, Crossover Controlled feeing study, 2-week run-in, 12 weeks DASH vs. Control arms each with 3 sodium levels for 4 weeks each intervention ¥	Adults with HTN, M + F, mixed race, *n* = 193 (control), *n* = 202 (DASH) from DASH-Sodium trial	LC-MS, untargeted	Urine	Identified several novel metabolite markers of the DASH diet; Replicated 8 significant urine metabolites identified in serum of original DASH trial that distinguish DASH vs. Control; Identified 9 significant urine metabolites identical in DASH-high sodium and DASH-low sodium diets
Pourafshar S, et al., 2021 Nutrients [92]	Characterize changes in metabolome following DASH diet intervention	RCT, Parallel, Controlled feeding study, 1 week Control run-in, 2 week feeding DASH diet or Control Ş	Adults with HTN, M + F, mixed race, *n* = 20	GC-MS, untargeted	Plasma and urine	Urine—19 metabolites differed significantly DASH vs. Control; Plasma—8 metabolites differed significantly DASH vs. Control; Major classes included phenolics, amino acid, organooxygen compounds, and cofactors

DASH diet and metabolomic analyses in the literature. Seven DASH feeding trials have assessed diet-responsive metabolites in various biosamples. Table 1 shows a summary of the subject population, biosamples, omic technological approach, study aim, study design and diet, the main study findings, and the study reference. Ş: Separate DASH-style diet trial specimens. ¥: Measurements from DASH–Sodium trial specimens. ≠: Measurements from original DASH trial specimens. Abbreviations: J Card Fail—Journal of Cardiac Failure; AJCN—American Journal of Clinical Nutrition; Sci Report—Scientific Reports; Hypertension—Journal of Human Hypertension; MNFR—Molecular Nutrition & Food Research; RCT—randomized controlled trial; M—male; F—female; GC-MS—gas chromatography-mass spectrometry; LC-MS—liquid chromatography-mass spectrometry.

## Data Availability

Not applicable.

## Appendix A

**Table A1:** Comparison of nutrient composition of DASH diets used in feeding intervention studies.

Study	Appel et al., *NEJM* 1997 [53]	Sacks et al., *NEJM* 2001 (Design: Svetkey et al., *J Am Diet Assoc* 1999) [21,22]	Lin et al., *J Am Diet* *Assoc* 2007 [97]	Hummel et al., *Hypertension* 2012 [94]
DASH diet name used in study	Combination diet	DASH diet at low, intermediate, and high sodium levels	Established recommendations + DASH diet (EST+DASH)	Sodium-restricted DASH diet (DASH/SRD)
Diet comparators	Control; Fruits-and-vegetables	Control at low, intermediate, and high sodium levels	Control; Established recommendations	None (DASH/SRD compared to baseline diet)
Energy level associated with provided nutrient analyses (kcal/d)	2100	2100	1705	2040
Macronutrients				
Fat (% of total kcal)	26	26	26	29
Saturated fat (% of total kcal)	7	5	8	Not available
Monounsaturated fat (% of total kcal)	10	13	10	Not available
Polyunsaturated fat (% of total kcal)	7	8	6	Not available
Carbohydrates (% of total kcal)	57	56	57	58
Protein (% of total kcal)	18	18	18	19
Cholesterol (mg/d)	151	150	205	211
Fiber (g/d)	Not available	32	22	34
Micronutrients				
Sodium (mg/d)	2859	1150 or 2300 or 3450	2412	1450
Potassium (mg/d)	4415	4700	3256	4274
Magnesium (mg/d)	480	500	311	Not available
Calcium (mg/d)	1265	1250	907	1350
Nutrient values based on:	Menu nutrient analyses	Menu nutrient targets described in study design publication	Mean intakes calculated from 24-h recall data at 18-month timepoint in intervention. Energy based on absolute intake (unadjusted)	Mean menu nutrient analyses from days 16-21 of the study
Intervention duration	8 weeks	30 days	18 months	3 weeks
Participants	*N* = 459, adults with systolic BP < 160 mmHg and diastolic BP ranging 80–95 mmHg, 225 females and 234 males	*N* = 412, adults with BP > 120/80 mmHg, ~57% females and ~43% males	*N* = 810, prehypertensive adults not taking antihypertensives, ~60% females and ~40% males	*N* = 14, treated hypertension, compensated HFpEF, 13 females and 1 male
Mean BP changes in DASH diet group compared to control (mmHg)				Ambulatory 24-h BP compared to baseline
Systolic (mmHg)	−5.5	−5.9 high; −5.0 intermediate; −2.2 low	−4.6 at 6 months; −2.1 at 18 months	−7
Diastolic (mmHg)	−3.0	−2.9 high; −2.5 intermediate; −1.0 low	−2.1 at 6 months; −1.0 at 18 months	−5

## Appendix B

**Table A2:** Metabolite markers and relationship to DASH diet components.

Study	Omic Platform	Bio-Sample	Metabolites	Associated Foods/Food Components
[83]	Targeted University of Michigan Metabolomics Center GC/MS + LC/MS	Serum (DASH style diet)	Short-chain acyl carnitine metabolites increased significantly, L-carnitine and propionyl carnitine correlated with improved left ventricular cardiac function	Results associated with dietary changes and energy utilization; no specific foods were discussed other than increased fiber intake
[77]	Untargeted Metabolon, Inc (Durham, NC) UHPLC/MS + GC/MS	Plasma (DASH-Sodium trial specimens)	Sodium intake significantly associated with changes in 6 metabolite pathways. Adjustment for change in diet pattern or blood pressure did not alter associations. Significant pathways (most increased with sodium restriction, while γ-glutamyl decreased with sodium restriction): Fatty acid Food component or plant group Benzoate metabolism Methionine metabolism Tryptophan metabolism γ-glutamyl amino acid	Greatest responder to reduced sodium intake was increase in 4-ethylephenylsulfate (Benzoate pathway) gut metabolite associated with soy intake; 4-allylpheol sulfate & homostachydrine (food component or plant group metabolites) may be related to herbs and seasonings added to low sodium diet to increase palatability; decrease in isovalerate (fatty acid pathway) may be gut microbiota changes related to salt intake
[76]	Untargeted Metabolon, Inc (Durham, NC) UHPLC/MS + GC/MS	Serum (original DASH trial specimens)	Multiple metabolites differed significantly DASH vs. Control (97) and DASH vs. fruit/veg (67); metabolites represented classes of lipids, amino acids, xenobiotics and food components, cofactors and vitamins, carbohydrates 10 candidate biomarkers for assessing adherence to DASH diet: N-methylproline Stachydrine (proline betaine) Tryptophan betaine Methyl glucopyranoside β-cryptoxanthin Theobromine 7-methylurate 3-methylxanthine 7-methlyxanthine Chiro-inositol	Citrus fruits (N-methylproline and stachydrine) Legumes (Tryptophan betaine) Cereals and cereal products (glucopyranoside) Total fruits (Methyl-β-glucopyranoside) Carotenoid-rich fruits and vegetables (β-cryptoxanthin) Caffeine and chocolate (theobromine, 7-methylurate, 3- and 7-methylxanthine) Fruit, beans, grains, nuts, seeds (chiro-inositol, phospholipids)
[81]	Untargeted University of Colorado RP HPLC/TOF MS, ESI negative and positive	Urine & food (DASH style trial)	Food specific compounds from DASH diet containing 12 specified foods were detected in urine along with other metabolites (13-190 metabolites per food); 16 urinary metabolites were significantly associated with BP and 6 with change in BP: 3-indolebutyric acid 1-beta-D-Ribofuranosyl)-1,4-dihydronicotinamide Kynuramine Physoperuvine 2 unidentified compounds (mass of 265.0971 and 157.0373)	12 specific foods from a DASH diet and their metabolites were characterized in this study: Apples and apple juice, beef, blueberries, broccoli, chicken, coffee, cucumber, grapefruit, peanut butter, pork, tilapia
[79]	Untargeted West Coast Metabolomics Center, University of California Davis CHS LC/QTOF MS, ESI positive, ESI negative	Plasma (DASH-Sodium trial specimens, control diet only)	Baseline comparisons exhibited no differences, but 3 metabolites (2 lipids and one organic acid derivative) differed significantly in salt sensitive subjects with change in sodium intake: Tocolpherol alpha 2-ketoisocaprioic acid Citramalic acid	Salt sensitive individuals exhibit an altered metabolome on high salt intake, no specific foods or food components were investigated
[78]	Untargeted Metabolon, Inc (Durham, NC) RP UHPLC/MS ESI positive early and late, ESI negative, Hydrophilic UHPLC/MS ESI negative	Urine (DASH-Sodium trial specimens)	Identified additional novel urinary metabolites associated with DASH diet: N-methylglutamate 3,5-dihydroxybenzoic acid Phloroglucinol sulfate Galactonate Replicated 8 significant urine metabolites identified in serum of original DASH trial distinguishing DASH vs. Control; and 9 significant urine metabolites identical in DASH-high and low sodium diets. Most Influential DASH metabolites: N-methylproline Stachydrine (proline betaine) Chiro-inositol Theobromine Tryptophan betaine 3-methylxanthine	Plant foods (N-methylglutamate) Whole grains and cereals (3,5-dihydroxybenzoic acid) Beans and lentils (Phloroglucinol sulfate) Dairy, pulses and seeds (Galactonate)
[82]	Untargeted GC/MS Duke University	Plasma & urine (DASH style trial)	Urine—19 metabolites (phenolic acids and microbial metabolites) differed significantly DASH vs. Control; Plasma—8 metabolites differed significantly DASH vs. Control; Major classes included phenolics, amino acid, organooxygen compounds, and cofactors	Whole grains, fruits, vegetables, herbs (polyphenol and phenolic acid rich foods) Protein sources, legumes (amino acids, organooxygen, keto acid derivatives, quinolones)
